# Distinct Oligomerization of Lactic Acid in Aqueous Microdroplets

**DOI:** 10.1021/acs.jpca.5c04592

**Published:** 2025-10-29

**Authors:** Tarun Kumar Roy, Shu Yang, Meng Li, Satish Kumar, Cari S. Dutcher, Vicki H. Grassian

**Affiliations:** a Department of Chemistry and Biochemistry, 8784 University of California San Diego, La Jolla, California 92093, United States; b Department of Mechanical Engineering, 5635 University of Minnesota, Minneapolis, Minnesota 55455, United States; c Department of Chemical Engineering and Materials Science, 5635 University of Minnesota, Minneapolis, Minnesota 55455, United States

## Abstract

Lactic acid (LA), a fundamental building block for poly­(lactic acid) (PLA) and a key component of atmospheric organic aerosols, undergoes self-esterification to form oligomers in aqueous environments. While this process has been well studied in bulk solutions, its reaction mechanism in aqueous microdroplets under ambient conditions remains poorly understood. Here, we investigate the reaction of LA in single microdroplets under temperature- and relative humidity (RH)-controlled conditions using *in situ* confocal micro-Raman spectroscopy. A reaction–evaporation model incorporating surface and bulk reaction pathways, evaporation, and water partitioning quantitatively reproduced the observed reaction kinetics. Both the model and experimental results show that LA undergoes rapid intermolecular esterification, forming oligomers primarily consisting of trimers, tetramers, and pentamers. This oligomerization in microdroplets proceeds 3 orders of magnitude faster than that in bulk solutions, leading to pronounced size-dependent kinetics and a distinct thermodynamic equilibrium. Furthermore, comparison of lactic acid and pyruvic acid (PA) condensation reactions reveals that the kinetic behavior of these reactions in aqueous microdroplets is primarily governed by the interplay between chemical reactivity and evaporation dynamics.

## Graphical abstract

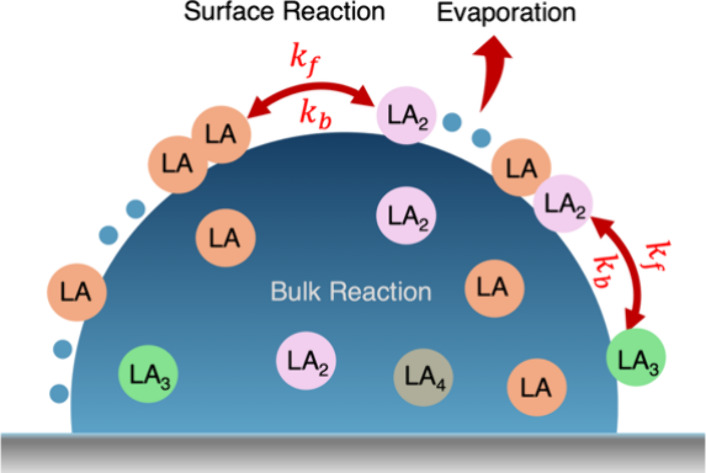

## Introduction

Microdroplets have been shown to enhance reaction rates by factors of one to 6 orders of magnitude relative to bulk solutions.^1^ ^–^ ^7^ This kinetic enhancement has been primarily attributed to the unique properties at the air–water interface of microdroplets^1–5^ and the fast gas-droplet partitioning.^6–8^ Notably, in condensation reactions, such as esterification reactions, thermodynamic and kinetic limitations due to water elimination in the bulk aqueous phase have been shown to be reduced at the microdroplet interfaces. At the interface, reactions are often closely coupled with adsorption and partitioning processes, complicating the understanding of the underlying mechanisms driving accelerated rates for these chemical reactions.^9^ Despite extensive studies on microdroplet kinetics, the role of interfacial physical processes remains poorly understood, and the thermodynamic behavior of reactions in microdroplets has received comparatively less attention.^10^

Lactic acid (LA), an α-hydroxy acid, serves as the fundamental building block in the synthesis of poly­(lactic acid) (PLA)—currently the most widely used biobased synthetic polymer.^11–13^ The production of PLA involves an initial step-growth condensation of lactic acid to form low-molecular-weight oligomers. These oligomers are then depolymerized into lactide, a cyclic dimer of lactic acid, which subsequently undergoes ring-opening polymerization (ROP) to produce high molecular weight PLA.^11^ Due to its biodegradability and sustainability features, PLA is extensively used in applications ranging from packaging and textiles to biomedical devices. The chemistry of lactic acid oligomerization is thus central to advancing sustainable materials and green polymer technologies.

In addition to its industrial relevance, LA is widely observed in atmospheric aerosols.^14–18^ It is a product of the oxidation of isoprene during secondary organic aerosol (SOA) formation^16^ and partitions between the gas phase and aerosol phase in the atmosphere.^14,15^ Due to its hydroxyl and carboxylic acid functional groups, LA can undergo intermolecular esterification in the aqueous phase to form oligomers. This process begins with the formation of a dimer (LA_2_), which can react with additional LA molecules to form higher oligomers such as trimers (LA_3_), tetramers (LA_4_), and beyond (Scheme 1).^19–21^ In aqueous solutions, the extent of oligomerization increases with a higher LA concentration and a lower water content.^20,21^ For example, when the weight percent of LA in water increases from 26.9 to 81.9%, the ratio of total oligomers to monomer increases from 0.038 to 0.304.^20^ Although LA can spontaneously oligomerize in aqueous solutions, the reaction is very slow at room temperature and usually requires elevated temperatures to increase the extent and rate of reaction.^20,22,23^ Note that heating a lactic acid solution prior to the reaction may hydrolyze oligomers back into monomers.^24,25^ Additionally, LA has been shown to evaporate more rapidly from aqueous salt aerosols than from bulk solutions.^26^ In aerosol microdroplets, the oligomerization of LA would strongly impact its gas-particle partitioning and SOA formation. However, the kinetics and thermodynamics of LA reactions in microdroplets have remained poorly characterized.

**Scheme 1:**
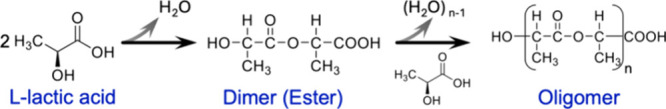
Self-Esterification of Lactic Acid (LA) to Form the Ester Dimer and Subsequent Oligomers^19–21^

In this study, we investigate the condensation reaction of LA in single microdroplets deposited on a hydrophobic coverslip in an environmental cell under relative humidity (RH)- and temperature-controlled conditions. Using *in situ* micro-Raman spectroscopy, we investigate the size- and RH-dependent reaction kinetics, along with the temporal evolution of microdroplet size. Furthermore, we determined the thermodynamic equilibrium distributions of LA and its oligomers across varying initial LA concentrations and compared these with bulk-phase behavior. To elucidate the role of interfacial physical processes governing reaction kinetics, we also present a detailed kinetic model combining the interfacial and bulk reactions with LA evaporation and water partitioning in microdroplets. The combination of experimental measurements and kinetic modeling reveals how the composition and size of microdroplets evolve as the reaction proceeds. This provides insights into how microdroplet reactivity is influenced by the coupling of interfacial reactions with gas-phase partitioning of LA and water under varying environmental conditions. To investigate how the air–water interface of microdroplets influences reaction pathways, we compared lactic acid oligomerization in microdroplets and bulk solutions. This allows us to assess how microdroplet environments alter the kinetics and thermodynamics of this condensation reaction. Finally, we compare the kinetics of LA condensation with those of pyruvic acid (PA) to gain broader insights into the chemistry at the air/water interface and condensation reactions in aqueous microdroplets.

## Methods

### Experimental Methods

#### Chemicals

l-Lactic acid (product number L 1750*:* 98% purity, termed ″lactic acid” in this work) was purchased from Sigma-Aldrich. Co. Pyruvic acid (98%, extra pure, nitrogen flushed) and methanol (HPLC grade) were purchased from Thermo Fisher Scientific. Milli-Q water (Millipore Sigma, 18.2 MΩ) was used as the solvent to obtain different concentrations of lactic acid (LA) solution.

#### Micro-Raman Spectroscopy Analysis and Generation of Microdroplets

The LA reaction in microdroplets was monitored using a confocal Raman spectroscopy (Horiba, LabRAM HR Evolution) coupled to a relative humidity (RH)- and temperature-controlled environmental cell (Linkam, LTS 120), as previously described in detail in Li et al.^6^ The microdroplets were generated using a medical-grade vibrating mesh nebulizer (OMRON MicroAIR U100) from a 0.5 mol kg^–1^ LA solution, and within less than 10 s, it was deposited on a hydrophobic coverslip prepared and then placed inside the environmental cell. After this step, the focus of the Raman laser was adjusted on the droplet, and a spectrum was collected within another ∼1.5 min. The measured contact angles of the droplets were typically 92–94°, which supports the fact that the droplets are hemispherical in our measurements. To prepare the hydrophobic coverslip, a quartz plate was dip-coated in a Rain-X solution and then air-dried. The optical images and Raman spectra of a single microdroplet were collected *in situ* during the microdroplet reaction utilizing a 100x super long working distance objective and a 532 nm laser. The size of the microdroplet was determined with the optical microscope with an uncertainty of ±1 μm. The confocal Raman system employed in this study provided a spectral resolution of ca. 1 cm^–^^1^, ensuring a clear distinction of characteristic vibrational modes. The RH within the environmental cell was controlled by high-purity nitrogen gas mixtures of dry and wet flows. The microdroplet was equilibrated with the water vapor within the cell. All experiments, except for those investigating the effects of RH, were conducted at 80% RH and 295 K under dark conditions.

#### Surface Tension Measurements

Surface tension measurements were carried out using a Kibron AquaPi tensiometer with Teflon sample cups. The tensiometer was calibrated with a milli-Q water to 72.8 ± 0.1 mN m^–1^. For each concentration, three independent solutions were prepared, each containing 7 mL of sample. The surface tension of each solution was measured three times, and the average value for each solution was calculated. The reported surface tension at each concentration corresponds to the overall average of the three solutions.

#### Mass Spectrometric Analysis

The products from the LA microdroplet reaction were analyzed using a direct-injection linear ion trap (Thermo Fisher Orbitrap) high-resolution mass spectrometer (HRMS). The products were prepared by collecting PA microdroplets on hydrophobic coverslips. The hydrophobic coverslips with PA microdroplets were placed in the Raman environmental cell at 295 K and 80% RH. After the completion of the reaction was confirmed by Raman spectra, the microdroplets containing products were dissolved in methanol for HRMS analysis in positive electrospray ionization (ESI) modes. Parameters for the heated ESI source are as follows: heater temperature, 120 °C; spray voltage, 3.00 kV; capillary temperature, 300 °C.

#### Kinetic Model

We developed a reaction–evaporation model for a hemispherical droplet of radius *R*, containing lactic acid (LA) and its oligomers (dimer through pentamer), which undergo oligomerization reactions both at the surface and in the bulk. The oligomerization reactions can be summarized as^27^

$$A(P_{m})B+A(P_{n})B\overset{k_{f}}{\underset{k_{b}}{⇌}}A(P_{m+n})B+H_{2}O$$ 1

where A and B represent carboxylic acid and hydroxyl groups, respectively, and P denotes the repeating unit. *k*_*f*_ and *k*_*b*_ are the forward and backward reaction rate constants. The subscripts *m* and *n* indicate the chain lengths of the reacting molecules. Experimental observations indicated that oligomers smaller than pentamers were the predominant species (Figure 1b). Consequently, the reaction scheme in the model includes oligomerization and dissociation processes among the species M_1_ (LA) through M_5_, with M_*n*_ representing an *n*-mer:

$$\begin{array}{c} M_{1}+M_{1}\overset{k_{f,j}}{\underset{k_{b,j}}{⇌}}M_{2}+H_{2}O\\ M_{1}+M_{2}\overset{k_{f,j}}{\underset{k_{b,j}}{⇌}}M_{3}+H_{2}O\\ M_{2}+M_{2}\overset{k_{f,j}}{\underset{k_{b,j}}{⇌}}M_{4}+H_{2}O\\ M_{1}+M_{3}\overset{k_{f,j}}{\underset{k_{b,j}}{⇌}}M_{4}+H_{2}O\\ M_{2}+M_{3}\overset{k_{f,j}}{\underset{k_{b,j}}{⇌}}M_{5}+H_{2}O\\ M_{1}+M_{4}\overset{k_{f,j}}{\underset{k_{b,j}}{⇌}}M_{5}+H_{2}O \end{array}$$ 2

Here, *j* in *k*_*f*, *j*_ and *k*_*b*, *j*_ refers to either surface (*j* = *s*) or bulk (*j* = *b*) reactions.

**Figure 1:**
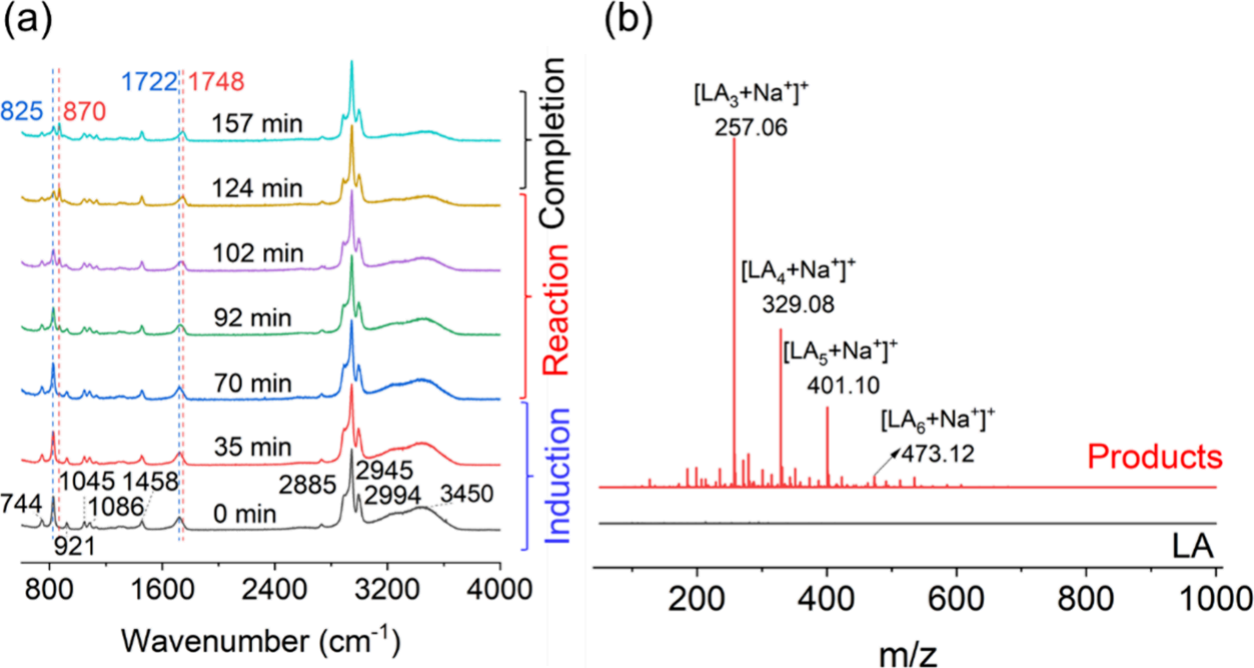
Oligomerization of lactic acid (LA) in microdroplets. (a) Micro-Raman spectra of a single aqueous microdroplet containing LA with an initial radius (*R*_o_) of 38 μm as a function of time from *t* = 0 to *t* = 157 min. The initial LA concentration in the microdroplet is 20 mol kg^–1^. (b) Mass spectrum of LA and its products in microdroplets collected in positive-ion mode. The LA reaction in microdroplets was performed at 80% RH and 295 K.

Negligible radial concentration gradients of LA were observed (Figure S1), suggesting that diffusion is not a rate-limiting process in microdroplets. Adsorption is neglected due to the low surface affinity of LA, leading to equal surface and bulk concentrations. Adsorption is neglected in the model because its characteristic time scale is substantially shorter than those of reaction or evaporation (see the SI). For simplicity, surface and bulk concentrations are assumed to be equal, where the surface concentration is simply approximated as the outermost 1 nm-thick layer of the bulk phase. However, surface tension measurements (Figure S2) suggest that a surface concentration based on the surface excess concentration applied to a 1 nm-thick region may be different than the value used in the model. This discrepancy in the surface to bulk concentration would result in a corresponding change of the reaction rate coefficients, although the deviation is not expected to exceed an order of magnitude (see the estimation in SI Adsorption kinetics). LA is volatile, while oligomers are assumed nonvolatile. Water partitioning is considered under constant RH. The concentrations of LA and its oligomers are denoted as *c*_*i*_(*t*), where *t* is time and *i* = 1 to 5 corresponds to the monomer (LA, *i* = 1), dimer (*i* = 2), trimer (*i* = 3), tetramer (*i* = 4), and pentamer (*i* = 5). Concentrations are expressed in molarity *c*_*i*_ (moles per cubic meter of solution) here and later converted to *m*_*i*_ (moles per kilogram of water) for comparison with experimental data. The primary nomenclature in the derivation is summarized in Table 1. Based on mass conservation, the change in the total moles of species *i* in the droplet is given by

$$\frac{d}{dt}\left(\frac{2}{3}\pi R^{3}c_{i}\right)=2\pi R^{2}\delta J_{i,s}+\frac{2}{3}\pi R^{3}J_{i,b}+J_{i,evp}$$ 3

**Table 1:** Primary Nomenclature in Derivation

symbol	nomenclature	units
*c*_*i*_	ratio of moles of solute to the volume of solvent	mol m^–3^
*m*_*i*_	ratio of moles of solute to per kilogram of water	mol kg^–1^
*V*_*i*_	volume	m^3^
*v*_*i*_	molar volume	m^3^ mol^–1^
*J*_*i*, *j*_	volumetric reaction rate	mol m^–3^ s^–1^
*J*_*i*, *evp*_	molar evaporation rate	mol s^–1^

The equation represents the contribution from surface reactions, where δ = 1 nm denotes the thickness of the reactive surface layer. The second term accounts for bulk reactions. Here, *J*_*i*, *s*_ and *J*_*i*, *b*_ are the volumetric reaction rate of species *i* from surface and bulk reactions, respectively. The third term represents the contribution from evaporation. *J*_1, *evp*_ denotes the rate of LA loss via evaporation and is defined in eq 5. For *i* > 1, *J*_*i*, and *evp*_ = 0 as oligomers are assumed nonvolatile.

The rate change of *c*_*i*_ due to reactions, represented by *J*_*i*, *j*_ multiplied by the reaction volume, is calculated from (2), where *j* refers to either surface (*j* = *s*) or bulk (*j* = *b*) reactions

$$\begin{array}{c} J_{1,j}=-2k_{f,j}c_{1}^{2}-k_{f,j}c_{1}c_{2}-k_{f,j}c_{1}c_{3}-k_{f,j}c_{1}c_{4}+2k_{b,j}c_{2}+k_{b,j}c_{3}+k_{b,j}c_{4}+k_{b,j}c_{5}\\ J_{2,j}=k_{f,j}c_{1}^{2}-k_{f,j}c_{1}c_{2}-2k_{f,j}c_{2}^{2}-k_{f,j}c_{2}c_{3}-k_{b,j}c_{2}+k_{b,j}c_{3}+2k_{b,j}c_{4}+k_{b,j}c_{5}\\ J_{3,j}=k_{f,j}c_{1}c_{2}-k_{f,j}c_{1}c_{3}-k_{f,j}c_{4}c_{3}-k_{b,j}c_{3}+k_{b,j}c_{4}+k_{b,j}c_{5}\\ J_{4,j}=k_{f,j}c_{2}^{2}+k_{f,j}c_{1}c_{3}-k_{f,j}c_{4}c_{1}-2k_{b,j}c_{4}+k_{b,j}c_{5}\\ J_{5,j}=k_{f,j}c_{1}c_{4}+k_{f,j}c_{2}c_{3}-2k_{b,j}c_{5} \end{array}$$ 4

Here, *k*_*f*, *j*_ and *k*_*b*, *j*_ are reaction rate coefficients for forward and backward reactions, respectively. The reaction rates are assumed to be independent of the degree of polymerization of the reactant.

The concentrations (eq 3) are solved alongside a size-evolution model that describes the time-dependent droplet radius *R*(*t*), influenced by LA evaporation and water partitioning. The size-evolution model follows the formulation developed in our previous studies, where further details can be found. For gas-phase diffusion-controlled evaporation, the rate of total moles of LA lost is^28,29^

$$J_{1,evp}=-2\pi RD_{LA}x_{1}P_{sat,LA}k_{evp}$$ 5

Here, *D*_*LA*_ is the diffusivity of LA in the gas phase, *x*_1_ is the mole fraction of LA at the surface, and *P*_*sat*, *LA*_ is the saturation pressure of LA at 295 K. Table 2 presents the values of the parameters used in the governing equations. The dimensionless correction factor *k*_*evp*_ in (5) accounts for discrepancies between the modeled and experimental evaporation rates. Since the activity coefficient of LA and other nonideal effects are unknown, *k*_*evp*_ is treated as a fitted parameter. In the results presented here, *k*_*evp*_ = 0.4. In the size-evolution model, molar fluxes are converted to volume (*V*) changes for the sake of convenience. Accordingly, the volume change of lactic acid (LA) due to evaporation is given by

$$\frac{dV_{1,evp}}{dt}=J_{1,evp}\nu_{1}$$ 6

where ν_1_ is the molar volume of LA, denoting the volume of LA per mole.

**Table 2:** Values of Quantities Used in the Kinetic Model^a^

parameter	value	units
*P*_*sat*, *LA*_	2.67	Pa
*D*_*LA*_	7.2 × 10^–6^	m^2^ s^–1^
ν_1_	7.51 × 10^–5^	m^3^ mol^–1^
ν_2_	1.28 × 10^–4^	m^3^ mol^–1^
ν_3_	1.75 × 10^–4^	m^3^ mol^–1^
ν_4_	2.15 × 10^–4^	m^3^ mol^–1^
ν_5_	2.50 × 10^–4^	m^3^ mol^–1^

a The molar volume of LA (ν_1_) is obtained from ref 30, where ν_*i*_ (*i* > 2) are estimated using the molecular weight from ref 31 divided by the same density of LA, assuming they have the same density. The estimations for *P*_*sat*, *LA*_ and *D*_*LA*_ are detailed in the SI.

Additionally, the change in volume of species *i* resulting from reactions is

$$\frac{dV_{i,rxn}}{dt}=\left(2\pi R^{2}\delta J_{i,s}+\frac{2}{3}\pi R^{3}J_{i,b}\right)\nu_{i}$$ 7

Because the saturation vapor pressure of water is approximately 300 times greater than that of LA, the partitioning behavior of water in microdroplets is primarily dictated by the water content. Although hydrogen-bonding interactions with LA and its oligomers, as well as desorption of LA from the interface, could influence water partitioning, the measured m_LA_ remains relatively constant during the induction stage (Figure 2a,c), even as the droplet radius decreases (Figure 4). This indicates that rapid equilibration of the water content is achieved during this phase, thereby supporting the validity of our assumption under the reaction conditions examined. So at a given RH, the change of one mole of species *i* corresponds to a change of *k*_*i*_ moles of water, leading to the relation of d*V*_*W*_/d*V*_*i*_ = *k*_*i*_ν_*w*_/ν_*i*_. Consequently, the water content change resulting from the X content change is

$$\frac{dV_{i}}{dt}=\frac{dV_{i}}{dt}\frac{k_{i}\nu_{w}}{\nu_{i}}$$ 8

**Figure 2:**
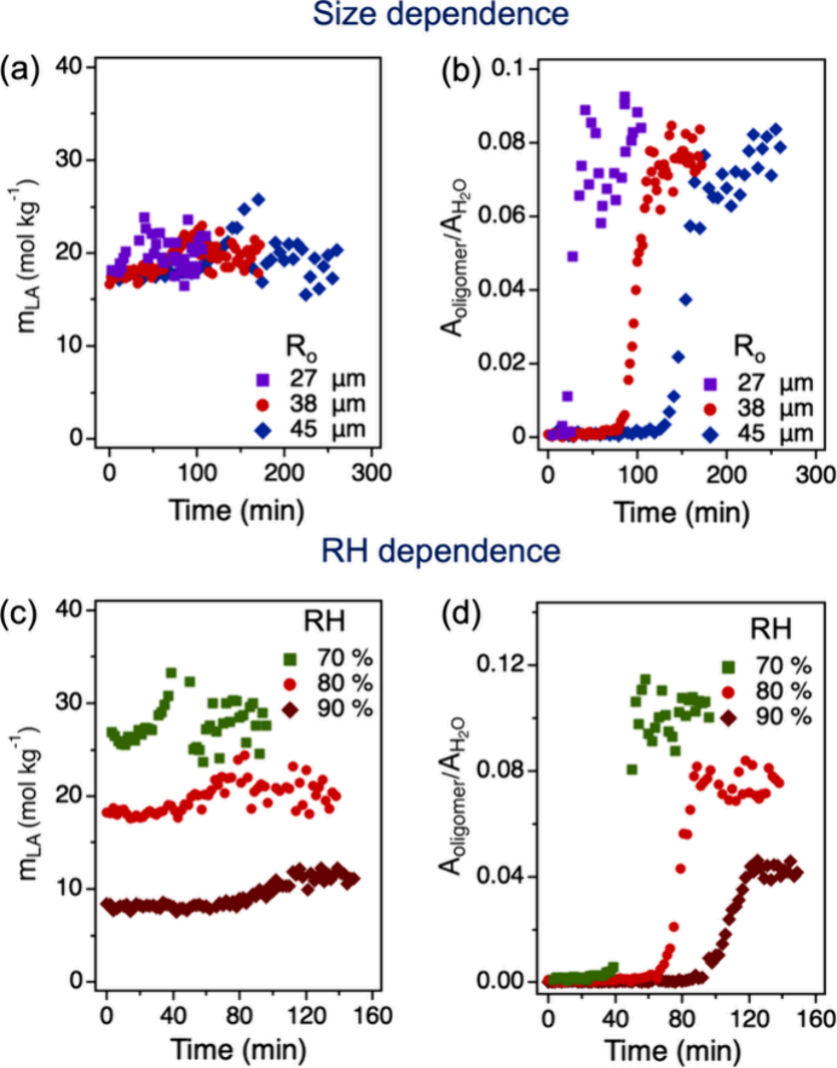
Size- and RH-dependent kinetics of LA oligomerization in microdroplets. Time evolution of the concentration of LA, *m*_LA_ (a), and the integrated Raman peak area ratio of the oligomers (ν­(C=O) at 1748 cm^–1^) and H_2_O (OH band from 3180 to 3750 cm^–1^, *A*_oligomer_/*A*_H_2_O_ (b), for droplets with varying initial radii (*R*_o_) at 80% RH and 295 K. Time evolution of *m*_LA_ (c) and *A*_oligomer_/*A*_H_2_O_ (d) at different RHs and 295 K in droplets with radii of 36 ± 1 μm.

At RH = 70%, *k*_1_ = 3.2 is estimated from the molality of LA obtained from the induction period with *m*_1_ = 17 mol kg^–1^. However, estimating *k*_*i*_ for *i* > 1 is more challenging and cannot rely on the aerosol inorganic–organic mixtures functional groups activity coefficients (AIOMFAC) model used in our previous work due to the substantial differences in hygroscopicity observed between LA and its oligomers. During the completion stage of the evaporation–reaction process, *m*_1_ remains nearly constant at a value close to its initial level despite the presence of produced oligomers. The water content should also remain unchanged, as it is in instantaneous equilibrium with the gas phase. Based on these observations, the final LA concentration and droplet radius suggest that approximately 3% of LA remains nonvolatile, retaining water in the droplet, while the oligomers absorb only a minimal amount of water. A value of *k*_i_ = 0.2 for *i* > 1 was obtained by fitting to the experimental data, with *k*_*evp*_ and the reaction rate coefficients fitted simultaneously to achieve the best overall agreement across all data sets.

With eqs 5–8, the total volume change of the droplet can be expressed as

$$2\pi R^{2}\frac{dR}{dt}=\frac{dV_{1,evp}}{dt}\left(1+\frac{k_{PA}\nu_{w}}{\nu_{PA}}\right)+\sum_{i=1}^{5}\frac{dV_{i,rxn}}{dt}\left(1+\frac{k_{i}\nu_{w}}{\nu_{i}}\right)$$ 9

The five concentration equations enclosed in eq 3, together with the droplet radius in eq 9, constitute the reaction–evaporation model. This model is solved numerically using MATLAB’s ode15s solver. The model simplifies the system by neglecting deviations from ideal hemispherical droplet geometry, as well as adsorption and diffusion processes, with the aim of capturing the reaction kinetics and quantitatively predicting experimental results using a minimal model. Note that although the water produced during the oligomerization reaction is not explicitly included in the equations, it affects the molar fraction and concentration of LA, which in turn influences both evaporation and reaction rates. Reaction rate constants are optimized by fitting the model to experimental data.

## Results and Discussion

### Size- and RH-Dependent Kinetics

Figure 1a presents the time-resolved Raman spectra recorded during the reaction of lactic acid (LA) in a microdroplet with an initial radius (*R*_o_) of 38 μm at 80% relative humidity and 295 K. The reaction exhibits a clear induction period (0 to 70 min) with minimal spectral changes, followed by a rapid reaction phase (70 to 124 min), and eventually reaching completion. This sequence of induction, reaction, and completion is similar to the condensation reaction observed in microdroplets for pyruvic acid.^6,7^ During the reaction period, four characteristic Raman peaks change substantially in intensity: (i) the peaks of LA at 825 cm^–1^ (*v*(C–COOH)) and 1722 cm^–1^ (*v* (C=O))^32–34^ decrease and (ii) the peaks of its oligomers (dimer and higher “mers”) at 870 cm^–1^ (*v*(C–COO^–^)) and 1748 cm^–1^ (*v*(C=O)^33,34^ increase. These spectral changes are indicative of the intermolecular esterification of LA to its oligomers in microdroplets. Additionally, the H_2_O band (*v*(OH)) centered at 3450 cm^–1^ decreases significantly along with the formation of oligomers, indicating the reduced hygroscopicity of the formed oligomers relative to LA. Other Raman peaks from low wavenumber to high wavenumber are δ­(OCO) at 744 cm^–1^, rocking of CH_3_ (r­(CH_3_)) at 921 cm^–1^, *v*(C–CH_3_) at 1045 cm^–1^, *v*(CO) at 1086 cm^–1^, δ­(CH_3_) at 1458 cm^–1^, and *v* (CH), *v*_asym_(CH_3_) and *v*_sym_ (CH_3_) at 2885, 2945, and 2994 cm^–1^.^32,33^ These peaks in the spectra do not change significantly, likely due to the overlap between LA and oligomer signals. Further confirmation of the formation of oligomers is obtained by high-resolution mass spectrometry. Figure 1b displays distinct mass peaks corresponding to lactic acid trimers (LA_3_), tetramers (LA_4_), and pentamers (LA_5_), confirming the presence of specific oligomeric products formed in the microdroplets.

To quantify the esterification kinetics, we monitored the time evolution of the concentration of LA and oligomers in microdroplets with varying initial droplet radius (*R*_o_), as shown in the top and bottom panels of Figure 2. The concentration of LA (*m*_LA_, in units of mol kg^–1^) with an associated uncertainty of ± 0.8 mol kg^–^^1^ during the droplet reaction was determined from its calibration curve relating *m*_LA_ to the integrated Raman peak area ratio of LA (ν­(C=O) at 1722 cm^–1^) and H_2_O (O–H band) (A _LA_/A_H_2_O_) (Figure S3). This ratiometric calibration method has been widely applied for accurate concentration determination of concentration in microdroplets.^35–37^ As shown in Figure 2a, *m*_LA_ remains nearly constant during the reaction at a fixed RH, suggesting that the hygroscopicity of the oligomers is very low and that the water absorbed by the newly formed oligomers is minimal. Due to the lack of standards for the oligomers, we used the peak area ratio of the oligomers (ν­(C=O) at 1748 cm^–1^) and H_2_O (OH band) (*A*_oligomer_/*A*_H_2_O_) to represent the total oligomer concentrations in droplets. The peak area ratio has been previously suggested to be a good choice to reflect the concentration, which is further validated by the similar distribution between *A*_LA_/*A*_H_2_O_ (Figure S4) and *m*_LA_. Note that the 1748 cm^–^^1^ peak is chosen as the oligomer peak rather than the 870 cm^–^^1^ peak because it exhibits a higher signal-to-noise ratio. As shown in Figure 2b, the formation of oligomers shows a size-dependent sigmoidal shape, with smaller droplets exhibiting both shorter induction times and a steeper increase in the level of *A*_oligomer_/*A*_H_2_O_. Consequently, the maximum apparent reaction rate, defined as the maximum slope of *A*_oligomer_/*A*_H_2_O_, is higher for smaller droplets. This behavior is similar to the reaction kinetics observed for the formation of zymonic acid through the condensation of pyruvic acid in microdroplets, where the size-dependent sigmoidal reaction kinetics were found to be driven by coupled surface reactions and gas-droplet partitioning of pyruvic acid.^6,7^ In the case of LA, its semivolatile nature, combined with the high surface-to-volume ratio of microdroplets, suggests that the gas-droplet partitioning and surface reaction may also play an important role in the observed kinetics.

The bottom panel of Figure 2 explores the effect of RH on LA oligomerization in microdroplets with an identical initial radius of 36 ± 1 μm. We observed a higher *m*_LA_ under a lower RH condition (Figure 2c). This is attributed to the rapid partitioning of water molecules between the droplet phase and the gas phase. A lower RH leads to a reduced water content in the droplet, thereby increasing m_LA_ within the microdroplet. From Figure 2d, it was observed that the oligomerization of LA occurs faster at a lower RH, suggesting that an increase in the m_LA_ enhances reaction kinetics. After the reaction completes and the system reaches equilibrium, the total oligomer concentration (represented by *A*_oligomer_/*A*_H_2_O_) reaches a stable value and shows a clear dependence on RH. These experimental results demonstrate that the extent of LA oligomerization in microdroplets and the associated reaction kinetics are strongly influenced by both droplet size and RH. To further investigate the underlying factors governing this complex behavior, we performed kinetic modeling of LA oligomerization at the air–water interface in aqueous microdroplets, as discussed below.

### Kinetic Modeling

We developed a reaction–evaporation model to elucidate the kinetics of LA reactions in aqueous microdroplets. The model provides a detailed kinetic framework in which oligomerization reactions occur both at the surface and in the bulk phase, with distinct reaction rates determined by experimental fitting. The bulk-phase reactions in microdroplets are described using the same rate constants as in bulk solution,^38^ whereas the markedly enhanced surface-phase rate constants account for the increased reactivity observed in microdroplets. These reactions are coupled with LA evaporation and water partitioning in a hemispherical microdroplet, while the resulting oligomers are treated as nonvolatile. Since the vapor pressure of water is much higher than that of LA, the water content in the droplet and the gas phase is assumed to be in equilibrium. Consequently, any change in LA (due to reaction or evaporation) and oligomer content results in a corresponding adjustment of water partitioning. Therefore, during the induction period, where only minimal amounts of oligomers are formed, m_LA_ remains effectively constant. Moreover, *m*_LA_ was observed to vary only slightly even during the reaction stage (Figure 2a,c), suggesting that the oligomers formed are only weakly hygroscopic. Accordingly, the uptake of water by the newly formed oligomers is assumed to be minimal in the model. Note that diffusion and adsorption are neglected in this model, as they have been found not to be rate-limiting steps in microdroplet reactions.^6–8^ This model enables the estimation of reaction rate coefficients through fitting to experimental data and provides insight into the mechanisms underlying the complex reaction kinetics. The full set of model equations is provided in the Methods section.

Figure 3 shows the kinetic modeling data using surface reaction rate coefficients, *k*_f, s_ = 6 × 10^–3^ kg mol^–1^ s^–1^, *k*_b, s_ = 3 × 10^–4^ s^–1^, and bulk reaction rate coefficients *k*_f, b_ = 1.8 × 10^–9^kg mol^–1^ s^–1^, and *k*_b, b_ = 9 × 10^–11^s^–1^. Alternative values of the reaction rate coefficients are tested in Figure S5; these yield poorer fits to the data but consistently confirm that surface reactions are much faster than bulk reactions. The same set of rate constants was applied to all simulations reported here, as they are independent of droplet radius and RH. These constants were obtained by fitting the kinetic model to experimental data. The surface reaction rate constants were primarily determined from the droplet composition data shown in Figure 2. The bulk reaction rate constants, obtained from bulk solution composition data in Figure 5, are much lower than the surface reaction rates and therefore make only a minor contribution to microdroplet kinetics, which is governed primarily by surface processes. A quantitative comparison of surface and bulk reaction time scales, together with their combined behavior in microdroplets, is provided in Table 3. Additionally, the time evolution of the droplet radius *R* (μm) during LA oligomerization (Figure 4) is used alongside concentration data to fit the reaction rate constants. Since the concentration of monomeric LA (*m*_LA_) changes only slightly over time, the model fitting primarily relied on the time evolution of the peak area ratio *A*_oligomer_/*A*_H_2_O_ (Figure 3b,d), and R (Figure 4). The value of oligomer concentration *m*_oligomer_ (mol oligomer per kg water) obtained from kinetic modeling does not quantitatively match the measured peak area ratio *A*_oligomer_/*A*_H_2_O_, since these quantities are only approximately related through a linear relationship by a calibration curve between these quantities. However, the evolution of *m*_oligomer_ from kinetic modeling shows consistent timing of the induction, reaction, and completion phases (Figure 3) compared with the experimental observations (Figure 2), indicating that the fitted reaction rate constants and model framework reliably capture the relative time scales of reaction and evaporation in microdroplets. Direct comparisons of the model and the experimental observations can be found in Figure S5 for different choices of reaction rate constants. Here, *m*_oligomer_ is the sum of the concentrations of M_2_ through M_5_. The contribution of each M_*i*_ to *m*_oligomer_ is shown in Figure S6, where shorter-chain oligomers are much more predominant. Accurately capturing the timing of these phases in the model requires reliable estimates of the reaction rate constants. Overall, the model quantitatively predicts the size dependence and RH dependence of the composition and droplet radius *R* throughout the reaction-evaporation process in the microdroplet.

**Figure 3:**
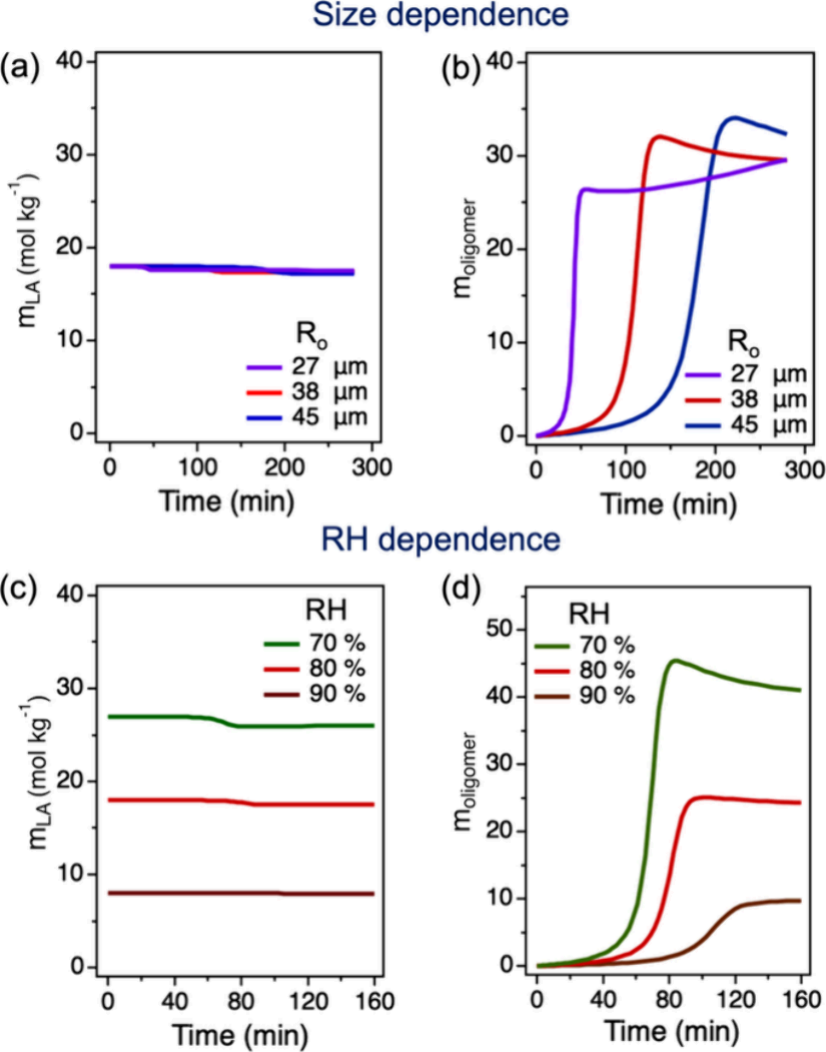
Kinetic modeling of LA oligomerization in microdroplets. The model incorporates LA evaporation, water (W) partitioning, and interfacial reactions leading to oligomer formation, along with a slow bulk reaction to best fit the experimental results. Time evolution of the concentration of LA, *m*_LA_ (a), and the concentration of oligomers *m*_oligomer_ (b), for droplets with varying initial radii (*R*_o_) at 80% RH and 295 K. Time evolution of *m*_LA_ (c) and *m*_oligomer_ (d) at different RHs and 295 K in droplets with *R*_o_ = 36 μm. The solid line represents kinetic modeling data using surface reaction rate coefficients *k*_f, s_ = 6 × 10^–3^ kg mol^–1^ s^–1^ and *k*_b, s_ = 3 × 10^–4^ s^–1^, and bulk reaction rate coefficients *k*_f, b_ = 1.8 × 10^–9^kg mol^–1^ s^–1^ and *k*_b, b_ = 9 × 10^–11^s^–1^.

**Figure 4:**
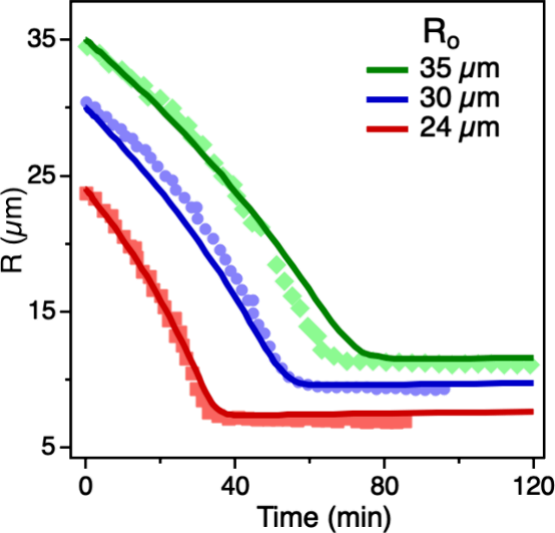
Time evolution of droplet radius during LA oligomerization at varying initial radii (*R*_o_). The droplet radius (*R*) was determined from bright-field images captured during micro-Raman spectroscopy. Reactions were performed at different *R*_o_ values at 80% relative humidity (RH) and 295 K, with an initial LA concentration of approximately 20 mol kg^–^^1^. The solid lines represent model fits to the experimental data points. Representative droplet images with a size of 30 ± 1 μm, obtained from the bright-field image in micro-Raman spectroscopy, are shown in Figure S7.

**Table 3:** Estimated Time Scale (min) of Surface Reactions (τ_*rxn*, *s*_), Bulk Reactions (τ_*rxn*, *b*_), and Total Reactions (τ_*rxn*_) for LA Oligomerization at Various Initial Droplet Radii *R*_o_ (Here, the Initial LA Concentration Is 17 mol kg^–^^1^)

*R*_o_	τ_*rxn*, *s*_	τ_*rxn*, *b*_	τ_*rxn*_
30 nm	8.2 × 10^–1^	2.7 × 10^5^	8.2 × 10^–1^
30 μm	8.2 × 10^2^	2.7 × 10^5^	8.2 × 10^2^
3 mm	8.2 × 10^4^	2.7 × 10^5^	6.3 × 10^4^
300 mm	8.2 × 10^6^	2.7 × 10^5^	2.6 × 10^5^

As observed in our previous study on the condensation reaction of pyruvic acid (PA), the profile of the product concentration (Figure 3b,d) and the microdroplet radius R (Figure 4) can be divided into three regions. During the induction period, *m*_oligomer_ increases slightly while R decreases, indicating that only minor reaction occurs, while LA evaporation and water partitioning drive the decrease of the droplet size. In the subsequent reaction period, *m*_oligomer_ sharply increases, and R decreases more rapidly, suggesting enhanced reactivity. The formation of dimers triggers further oligomerization, and the consumption of LA promotes additional water desorption, further accelerating the decrease in *R*. In the completion period, both *m*_oligomer_ and *R* level off as the reaction and evaporation slow down. However, unlike the pronounced decline in the PA concentration observed previously, *m*_LA_ remains nearly constant throughout the process (Figure 3a,c). This difference is likely due to the extremely low hygroscopicity of the LA-derived oligomers, which have minimal effect on the water partitioning. Additionally, LA evaporation appears to halt when approximately 2% of LA remains in the droplet. This may result from the formation of micelle-like structures or a dense surface layer of oligomers that inhibits further evaporation by creating a diffusion barrier. While the model predicts a slight decrease in *m*_LA_ and the experiment shows a slight increase, this discrepancy may stem from nonideal water–solute interactions and deviations in partitioning behavior not fully accounted for in the model.

### Aqueous Microdroplets versus Bulk Aqueous Solutions

To quantify the concentration ratio of oligomers to LA in microdroplets, we analyzed the peak area ratio of the carbonyl stretching modes: ν­(C=O) at 1748 cm^–^^1^ for oligomers and at 1722 cm^–^^1^ for LA (*A*_oligomer_/*A*_LA_). Figure 5a shows the RH dependence of this ratio for a fixed microdroplet size of 36 ± 1 μm. Kinetic modeling shows good agreement with the experimental time evolution, and the results indicate that the equilibrium oligomer-to-LA ratio at the completion stage of the reaction exhibits minimal variation with the RH.

**Figure 5:**
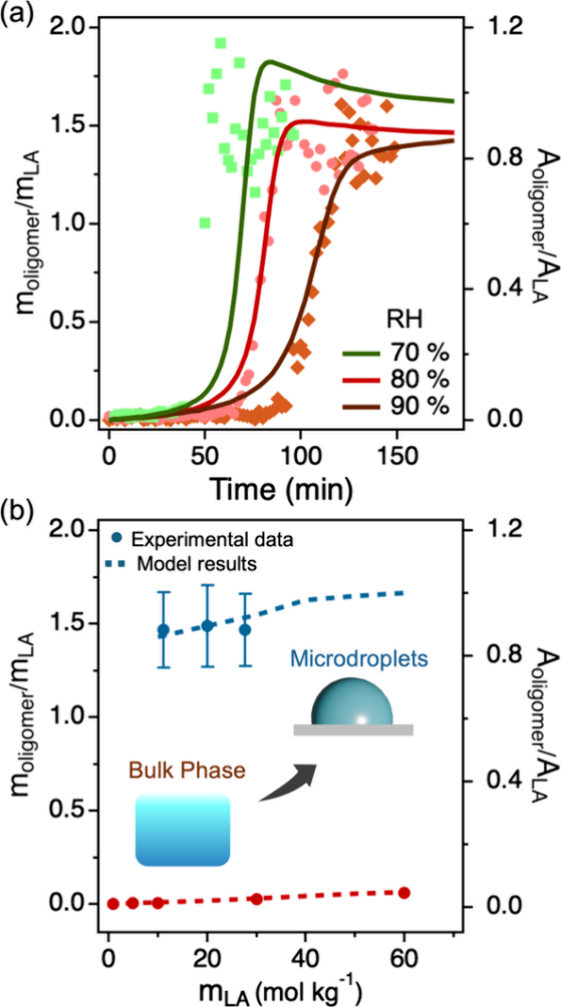
Distinct LA oligomerization in microdroplets versus bulk solutions. (a) Time evolution of the oligomer-to-LA concentration ratio (*m*_oligomers_/*m*_LA_, left *Y*-axis) in microdroplets at different RHs and 295 K at a consistent droplet size of 36 ± 1 μm and the corresponding peak area ratio (*A*_oligomers_/*A*_LA_, left *Y*-axis). (b) Changes in the equilibrium oligomer-to-LA ratio as a function of *m*_LA_ in microdroplets versus bulk solutions. The blue and red traces represent the microdroplet and bulk phases, respectively. In microdroplets, the equilibrium oligomer-to-LA ratio shows minimal variation with *m*_LA_, whereas in bulk solutions, the ratio increases with *m*_LA_. Dashed lines represent model fits to the experimental data points. Experimental data for bulk solutions with a reduced *Y*-axis scale are also provided in Figure S9 to better highlight subtle variations.

Although oligomerization is also detected in bulk LA solutions, the reaction is substantially slower (Figure S8). For example, after 7 days of reaction in solutions with initial LA concentrations ranging from 5 to 60 mol kg^–1^, fewer oligomers are formed compared to a microdroplet reacting for only 92 min (Figure 1a). It is worth noting that the droplet had an initial LA concentration of 20 mol kg^–1^, which is within the range of bulk concentrations. Therefore, the enhanced reactivity in microdroplets is not primarily from concentration differences between droplets and bulk solutions. The time scales of bulk reaction (τ_*rxn*, *b*_) and surface reaction (τ_*rxn*, *s*_) are estimated from the kinetic model using a scaling argument. Assuming that dimerization is the primary reaction and other reactions are negligible, the characteristic time scales can be approximated from eq 3 as follows:

$$\frac{\frac{2}{3}\pi R^{3}m_{\mathrm{LA}}}{\tau_{rxn,b}}∼2k_{f,b}m_{\mathrm{LA}}^{2}\frac{2}{3}\pi R^{3}$$ 10

$$\frac{\frac{2}{3}\pi R^{3}m_{\mathrm{LA}}}{\tau_{rxn,s}}∼2k_{f,s}m_{\mathrm{LA}}^{2}2\pi R^{2}\delta$$ 11

which results in

$$\tau_{rxn,b}=\frac{1}{2k_{f,b}m_{\mathrm{LA}}}$$ 12

$$\tau_{rxn,s}=\frac{R_{o}/\delta }{6k_{f,s}m_{\mathrm{LA}}}$$ 13

where δ = 1 nm is the thickness of the reacting surface. It is apparent that τ_*rxn*, *b*_ is independent of droplet size, while τ_*rxn*, *s*_ increases with droplet size. This is because bulk reactions scale with the volume of the droplet (*R* ^3^) for both reactant amount and reaction rate (eq 10), whereas surface reactions scale with surface area (*R* ^2^), but the reactant amount still depends on volume (eq 11). The estimated time scale for total reactions τ_*rxn*_ depends on both surface and bulk reactions:

$$\tau_{rxn}=\frac{1}{\frac{1}{\tau_{rxn,s}}+\frac{1}{\tau_{rxn,b}}}$$ 14

Using eqs 12–14, the values of τ_*rxn*, *s*_, τ_*rxn*, *b*_, and τ_*rxn*_ are calculated for a range of initial droplet radii *R*_o_, as presented in Table 3. The τ_*rxn*, *b*_ is independent of droplet size, whereas τ_*rxn*, *s*_ and τ_*rxn*_ increase with increasing droplet radius. For nano- and microsized droplets, the surface reaction dominates the total reaction time scale, whereas for larger droplets or bulk systems, the bulk reaction becomes more significant. While the reaction constants in microdroplets are approximately 7 orders of magnitude higher than those in the bulk phase (as discussed in the Figure 3), for a droplet with a radius of about 30 μm, the surface reaction time scale is about 3 orders of magnitude shorter than that of bulk reactions. This discrepancy arises because the confined geometry of the microdroplet surface limits the reactive area and available space limits the surface reaction (the *R*_o_/δ term in eq 13). Nevertheless, the significantly higher intrinsic reaction rates at the surface still make surface reactions much more dominant than those in the bulk phase. Moreover, the strong size-dependent kinetics observed in Figure 5a further indicate that surface reactions are significantly more dominant than bulk reactions.

We further compare the equilibrium oligomer-to-LA ratio (reflected by the experimental *A*_oligomer_/*A*_LA_ ratio) at varying initial LA concentrations (*m*_LA_) in microdroplets and bulk solutions, as shown in Figure 5b. Since *m*_LA_ in microdroplets is controlled by the surrounding RH, we varied the initial LA concentration by adjusting the RH in the environmental cell through mixing different ratios of dry and wet nitrogen gases. In the model, the microdroplet results are based on a 35 μm droplet with varying initial LA concentrations and corresponding water partitioning across different RH. The bulk results are based on a 5 mL liquid droplet, with the model assuming no surface reactions, evaporation, or water partitioning. The values of *m*_LA_ and corresponding equilibrium oligomer-to-LA ratios in microdroplets are obtained from Figure 5a. While the equilibrium oligomer concentration increases with the initial *m*_LA_ (Figures 2d and 3d), the equilibrium oligomer-to-LA ratio varies only modestly with initial *m*_LA_ and therefore shows little dependence on RH. This contrasts substantially with bulk-phase behaviors, where the thermodynamic equilibrium distribution of the oligomers to LA increases with increasing *m*_LA_ (Figure 5). Together, these findings demonstrate that LA undergoes a distinctly different and faster oligomerization process in microdroplets, driven by both accelerated surface kinetics and altered thermodynamic constraints compared to those of bulk solutions.

### Comparison between PA and LA Condensation Reactions in Aqueous Microdroplets

To gain a comprehensive understanding of the physicochemical factors governing condensation reactions of organic acids at the air–water interface, we compared the kinetics of lactic acid (LA) and pyruvic acid (PA) condensation in aqueous microdroplets. LA (α-hydroxy acid) and PA (α-keto acid) differ in the functional group at the α-carbon position: LA contains a hydroxyl group (−OH), whereas PA possesses a carbonyl group (C=O). This structural difference results in distinct chemical reactivity and condensation pathways.

As mentioned earlier, due to the presence of both hydroxyl and carboxylic acid functional groups, LA can undergo self-esterification in the aqueous phase, leading to the formation of oligomers. In contrast, PA condensation proceeds through its keto form, undergoing acid-catalyzed aldol addition followed by intramolecular cyclization to form zymonic acid (ZA).^6,7,38–40^ Parapyruvic acid was proposed as a reaction intermediate in the formation of ZA. Figure 6 presents the time evolution of product formation for LA and PA condensation reactions within aqueous microdroplets of initial radius (*R*_o_) 38 μm, maintained at 80% relative humidity and 295 K. The experimental data clearly show that oligomer formation from LA occurs at a significantly slower rate than that of ZA formation from PA, in agreement with kinetic modeling predictions. Note that both LA and PA exhibit sigmoidal reaction kinetics. While our previous model of PA demonstrated that sigmoidal concentration profiles can arise even in the absence of autocatalysis,^7^ the oligomerization of LA exhibits behavior similar to autocatalysis, where products accelerate the consumption of reactant and result in a sigmoidal concentration profile. Specifically, the formation of dimers promotes further oligomerization by consuming additional LA to form trimers and higher-order oligomers. This chain-like progression accelerates LA consumption, effectively mimicking an autocatalytic effect in which the products facilitate the faster consumption of the reactant.

**Figure 6:**
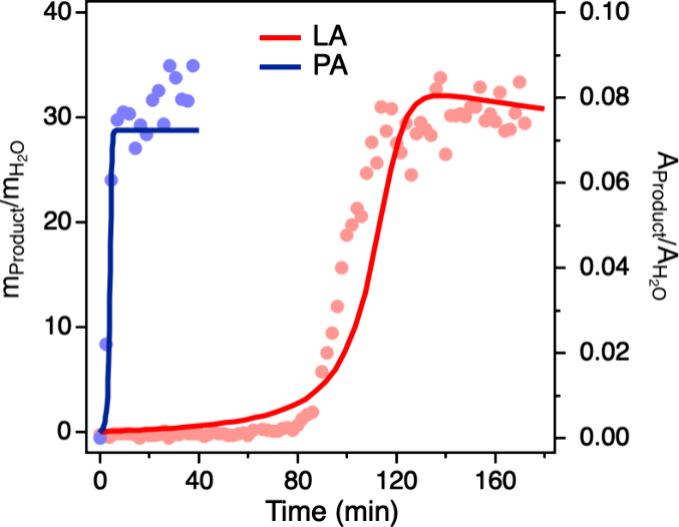
Comparison of the kinetics of PA and LA condensation reactions in microdroplets. The time evolution of product formation for LA and PA condensation reactions within aqueous microdroplets of initial radius (*R*_o_) 38 μm, maintained at 80% relative humidity and 295 K. The solid lines represent model fits to the experimental data points. Previous studies of pyruvic acid condensation in microdroplets were done with droplet radii from 70 to 700 μm, where for these larger droplets, the observed induction periods were more evident.^6,7^ New data with a smaller microdroplet with an initial radius of 38 μm is shown here to directly compare the time scales observed for these two different systems, but for the same-sized microdroplets.

As demonstrated in our previous studies, the time scales for diffusion and adsorption in microdroplets are orders of magnitude shorter than those for reaction and evaporation. We therefore propose that the observed differences in reaction kinetics mainly arise from three physicochemical factors influencing the condensation kinetics of LA and PA: (1) characteristic evaporation time scale (τ_*evp*_), (2) characteristic reaction time scale (τ_*rxn*_ defined in eq 14), and (3) the hygroscopicity of the resulting products. Here, τ_*evp*_ for LA is

$$\tau_{evp}=\frac{R_{o}^{2}}{E}x_{LA}$$ 15

where the evaporation coefficient *E* = *DP*_*sat*, *LA*_(ν_*LA*_ + *k*_*w*_ν_*w*_)/*R*_*g*_ *T*, and *x*_*LA*_ is LA mole fraction. Notably, the vapor pressure of LA *P*_*sat*, *LA*_ = 2.67 *Pa*, while that of PA is significantly higher, at 105 Pa, resulting in τ_*evp*_ of ca. 40 times faster for PA compared to LA. PA also exhibits a much lower τ_*rxn*_, the corresponding values of τ_*evp*_ and τ_*rxn*_ are listed in Table 4. Moreover, the oligomers formed from LA condensation are much less hygroscopic compared with ZA formed from PA condensation. As a result, the decrease in m_LA_ is relatively minor, since the oligomeric products have a much lower capacity to absorb water and dilute LA in the droplet. These differences collectively contribute to the slower reactivity of LA in microdroplet environments compared to that of PA.

**Table 4:** Estimated Time Scale of the Individual Process in LA and PA Condensation Reaction (*R*_o_ = 38 μm)

(mins)	τ_*evp*_	τ_*rxn*_
LA	2000	5000
PA	50	300

For both LA and PA reactions in microdroplets, adsorption and diffusion are not rate-limiting steps for evaporation or reaction. The characteristic diffusion time scale, τ_dif_ = *R* ^2^/*D*_*X*_ ≈ 10^–1^ min for PA and LA (with diffusivities of ca. 10^–9^ m^2^s^–1^), is much shorter than τ_*rxn*_ and τ_*evp*_, thereby justifying the assumption of instantaneous radial mixing. Given that the vapor pressure of water is much higher than that of LA or PA, the water content rapidly reaches equilibrium between the droplet and the gas phase (the time scale for water partitioning is assumed to be zero). This results in a near constant initial reactant concentration during the induction stage, where both the reactant and water evaporate simultaneously while the reaction remains minor. While the kinetics are dictated by the interplay between reaction and evaporation, a shorter τ_*rxn*_ leads to an earlier onset of the reaction stage and a sharper change in the concentration profile, whereas a shorter τ_*evp*_ results in faster completion of evaporation. Overall, the present studies show enhanced condensation kinetics of LA and PA at the air–water interface in aqueous microdroplets, which is consistent with prior findings of fast condensation reactions in microdroplets.^6,7,41–43^

## Conclusions

This study demonstrates that lactic acid undergoes markedly accelerated and thermodynamically distinct oligomerization through the self-esterification reaction in aqueous microdroplets compared to bulk solutions. Using *in situ* Raman spectroscopy and mass spectrometry, we show that LA rapidly forms trimers, tetramers, and pentamers in microdroplets. A reaction–evaporation model incorporating interfacial and bulk pathways, evaporation, and water partitioning quantitatively captures the observed kinetics and highlights the dominant role of surface reactions in microdroplet environments. More importantly, for the first time, we experimentally reveal that the esterification of LA in microdroplets follows distinct thermodynamics; i.e., the reaction favors the formation of oligomers, and it is not restricted to the concentration of monomer LA. Furthermore, comparison with pyruvic acid underscores that condensation kinetics in microdroplets are governed by a dynamic interplay between reaction rates, evaporation, and product hygroscopicity. These findings advance our understanding of the oligomerization reaction of LA at the air–water interface of aqueous microdroplets and have broad implications for atmospheric aerosol chemistry and the scalable synthesis of biopolymer precursors in industrial processes.

## Supplementary Material



## Notes

Additional information on the experiments and kinetic model, along with nine figures, including Raman spectra at different depths of the microdroplet (Figure S1); LA bulk concentration vs surface tension plot (Figure S2); calibration curve for LA concentration (Figure S3); time evolution of A_LA_/A_H_2_O_ (Figure S4); sensitivity analysis of reaction rate coefficients (Figure S5); distribution of oligomers (Figure S6); bright-field images of a microdroplet (Figure S7); Raman spectra of LA bulk solutions (Figure S8); and equilibrium oligomer-to-LA ratio as a function of *m*_LA_ in bulk solutions (Figure S9) (PDF)
