# Supplementary material for: Distinct Oligomerization of Lactic Acid in Aqueous Microdroplets
Source: J Phys Chem A. 2025 Oct 29;129(45):10540–9. doi: 10.1021/acs.jpca.5c04592 (PMC12621245; doi:10.1021/acs.jpca.5c04592)
Supplement: Supplementary file 1 [file jp5c04592_si_001.pdf]

Supporting Information

Distinct oligomerization of lactic acid in aqueous microdroplets

Tarun Kumar Roy‡[a], Shu Yang‡[b], Meng Li[a], Satish Kumar[c],
Cari S. Dutcher[c]* and Vicki H. Grassian[a]*

[a]Department of Chemistry and Biochemistry, University of California San Diego, La Jolla, California 92093, USA
[b]Department of Mechanical Engineering, University of Minnesota, Minneapolis, MN 55455, USA.
[c]Department of Chemical Engineering and Materials Science, University of Minnesota, Minneapolis, MN 55455, USA

‡ These authors contributed equally.
*Email: vhgrassian@ucsd.edu (V.H.G.) and cdutcher@umn.edu (C.S.D.)

This file includes additional information on the kinetic model and experiments, 9 figures (Figures S1 to S9) and references. The figures include: Raman spectra at different depth of the microdroplet (Figure S1); LA bulk concentration vs surface tension plot (Figure S2); calibration curve for LA concentration (Figure S3); time evolution of A_{LA}/A_{H_2O} (Figure S4); sensitivity analysis of reaction rate coefficients (Figure S5); distribution of oligomers (Figure S6); bright-field images of a microdroplet (Figure S7); Raman spectra of LA bulk solutions (Figure S8); and equilibrium oligomer-to-LA ratio as a function of m_{LA} in bulk solutions (Figure S9).

Measurements of concentration gradients in individual microdroplets

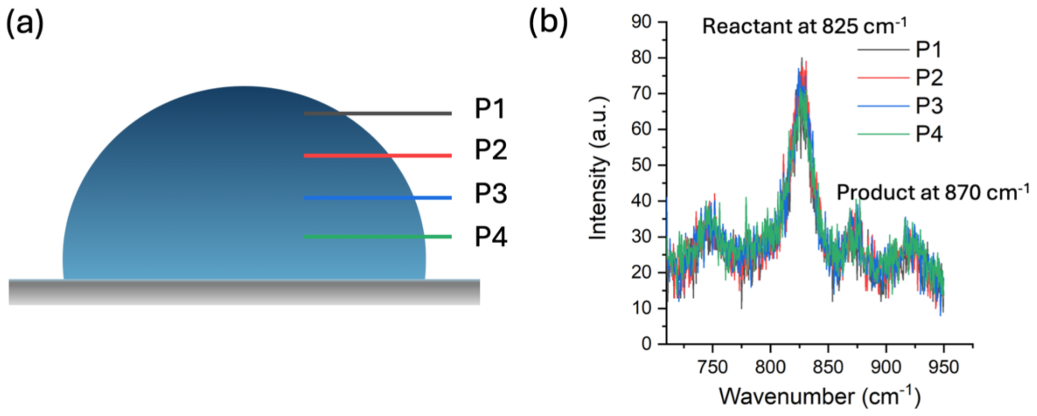

Figure S1: (a) Raman spectra were acquired from the center to the near-surface region of the microdroplet during product formation. (b) The nearly identical spectra indicate the absence of a significant concentration gradient for either the reactant or the product.

Adsorption kinetics

The adsorption kinetics of LA can be inferred from the dependence of surface tension (σ) on LA bulk concentration (c_1) (Figure S2), which can be described using a Langmuir adsorption (Szyszkowski equation):[1]

$$\sigma = \sigma_o + R_g T \Gamma_\infty \ln\left(1 - \frac{\kappa c_1}{1 + \kappa c_1}\right) \tag{SI-1}$$

where σ_o is the surface tension of pure water, R_g is the universal gas constant, and T=295 K. The adsorption equation in this form can be derived from the Gibbs adsorption equation for ideal solutions and the Langmuir isotherm:

$$\frac{\Gamma}{\Gamma_\infty} = \frac{\kappa c_1}{1 + \kappa c_1} \tag{SI-2}$$

which provides the fractional surface concentration (Γ/Γ_∞) as a function of the bulk concentration, where Γ_∞ is the maximum surface concentration, and κ is the adsorption equilibrium constant that characterizes the affinity of LA for the air–water interface. In the framework of Gibbs adsorption, the surface concentration Γ is the excess concentration ascribed to the hypothetical dividing surface relative to that of the solvent.[2] By fitting the measured surface tension data to Langmuir isotherm, we obtain $\Gamma_\infty = 2.55 \times 10^{-6}$ mol m^{-2} and $\kappa = 7.67 \times 10^{-3}$ m^{3}mol^{-1} for LA.

In Figure S2, we present LA concentration m_{LA} (mol kg^{-1}) as a function of σ and the relative surface adsorption Γ/Γ_∞. For the experimental LA concentrations employed in this study (8,

17, and 27 mol kg^{-1}), the surface concentrations approaches Γ_∞, and the bulk concentrations approach or exceed the concentration at which aggregation effects or saturation of interfacial sites become significant. In this regime, the Langmuir isotherm ceases to provide an accurate description, and the absence of adsorption data for oligomers further constrains the quantitative characterization of adsorption kinetics. Nevertheless, an order-of-magnitude estimate of the adsorption timescale:[3]

$$\tau_{\mathrm{ads}} = \frac{1}{\mathrm{k_d}(\kappa c_1 + 1)} \sim 1.90 \times 10^{-4} \quad \text{(SI-3)}$$

indicates that adsorption occurs on a timescale far shorter than that of reaction or evaporation (Table 3). It is therefore reasonable to neglect adsorption kinetics in the present analysis. Here $\mathrm{k_d}$ denotes the desorption rate constant, which exhibits minimal variation across different molecular structures and functional groups.[4] Accordingly, we adopt a representative value of $\mathrm{k_d} = 90$ s^{-1}.

An inaccuracy may arise in our model due to the assumption that the surface concentration is equal to the bulk concentration. To assess the magnitude of this error, we convert Γ from units of mol m^{-2} to bulk concentration units $\left(\text{mol m}^{-3}\right)$ by adopting a hypothetical surface thickness of 1 nm. To achieve the same surface reaction timescale $\tau_{rxn,s} = R_o/(\delta 6 k_{f,s} m_{LA})$ (Eq. 13), if the excess surface concentration is used as the reacting concentration at the surface, the surface reaction rate constants $k_{f/b,s}$ reported in the manuscript would be underestimated by a factor of five. If the total concentration (excess + bulk) is used as the reacting concentration at the surface, $k_{f/b,s}$ would be overestimated by approximately 17 %. Either way, the deviation in the estimated surface reaction rates introduced by this assumption is not expected to exceed an order of magnitude and, the surface reaction remains much faster than the bulk reaction.

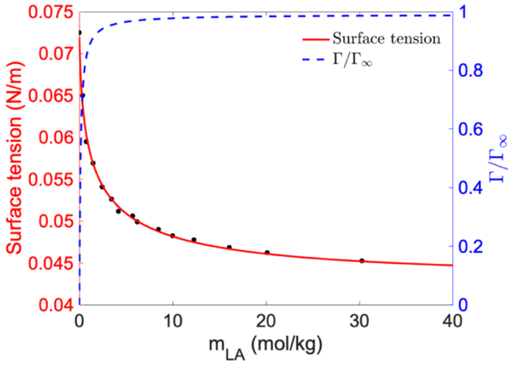

Figure S2: Relationship between LA bulk concentration m_{LA} and surface tension σ (left axis) and Γ/Γ_∞ (right axis). The black dots represent experimental data from surface tension measurement, while the red solid line corresponds to the Langmuir isotherm fit. The Γ/Γ_∞ calculated using Equation (SI-2) is shown as a blue dashed line.

Diffusivity and vapor pressure of LA

The gas-phase diffusivity of LA was calculated using the binary diffusion coefficient correlation developed by Fuller et al:[5]

$$D_{AB} = \frac{10^{-3} T^{1.75} \left(\frac{1}{M_A} + \frac{1}{M_B}\right)^{1/2}}{p[(\sum_A v_i)^{1/3} + (\sum_B v_i)^{1/3}]^2} \quad \text{(SI-4)}$$

Here, A and B denote LA and N_2, respectively. D_{AB} is the binary diffusion coefficient, T is temperature (K), M_A and M_B are the molecular weights, and p is the pressure. $\sum_A v_i$ represents the diffusion volume summed over the atoms of LA.

The vapor pressure of LA at 295 K (P_2) was estimated using the Clausius–Clapeyron relation, anchored by experimental vapor pressure (P_1):[6]

$$\ln\left(\frac{P_2}{P_1}\right) = -\frac{\Delta H_{\text{vap}}}{R}\left(\frac{1}{T_2} - \frac{1}{T_1}\right) \quad \text{(SI-5)}$$

where the enthalpy of vaporization of LA is $\Delta H_{\text{vap}} = 66$ kJ mol^{-1}, the vapor pressure at 295 K (T_2) is calculated from the experimental vapor pressure at 298 K (T_1).[6] The gas-phase diffusivity and vapor pressure of LA are listed in Table 2 in the main text.

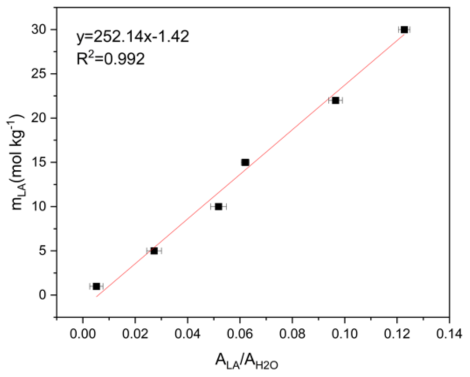

Figure S3. The calibration curve relating LA molality (m_{LA}) to the integrated Raman peak area ratio of LA (ν(C=O) at 1722 cm^{-1}) and H_2O (OH band from 3180 to 3750 cm^{-1}) (A_{LA}/A_{H2O}). Note, the LA peak at 825 cm^{-1} (ν(C-COOH)) was not used to calibrate and determine m_{LA}, because this peak overlaps with the ν(C-C) of the ester group in the oligomers.

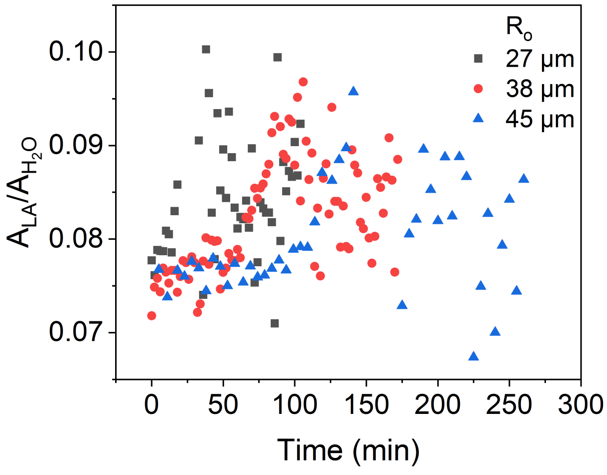

Figure S4. Time evolution of the integrated Raman peak area ratio of LA (ν(C=O) at 1722 cm^{-1}) and H_2O (OH band from 3180 to 3750 cm^{-1}) (A_{LA}/A_{H_2O}).

Sensitivity analysis of reaction rate coefficients

We performed a sensitivity analysis in which the surface and bulk reaction rate coefficients were varied over a wide range. As shown in Figure S5, alternative parameter choices yield poorer agreement with the experimental data but consistently confirm that the surface reactions proceed much faster than the bulk reactions. A faster or slower reaction rate (Figure S5b and S5c, respectively) shifts the timing of the induction, reaction, and completion phases away from experimental observations, while a higher bulk reaction rate reduces the size dependence of the kinetics (Figure S5d). This robustness indicates that, although the exact fitted values of the coefficients may vary within some range, the central conclusion of this work—that surface reaction rates exceed bulk reaction rates by orders of magnitude—remains unaffected.

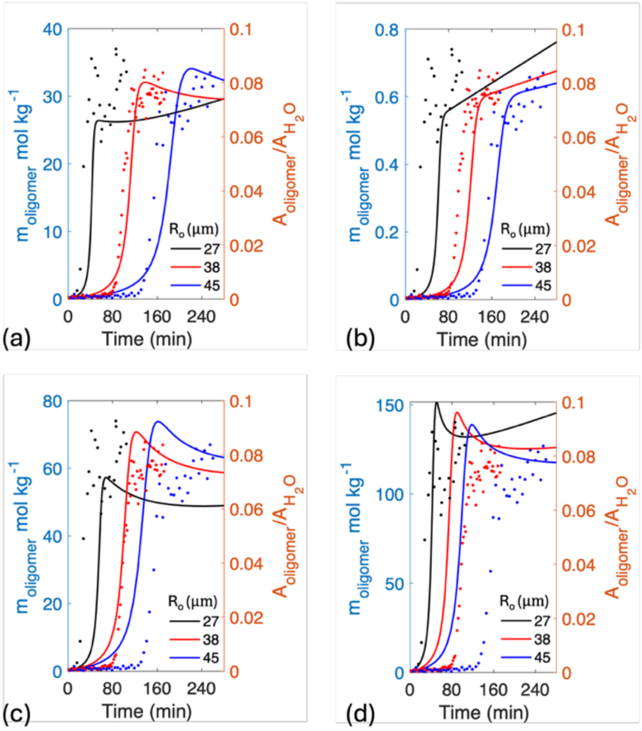

Figure S5: Time evolution of $A_{oligomer}/A_{H_2O}$ *(right axis) and* $m_{oligomer}$ *(left axis)* for droplets with varying initial radii (R_o) at 80% RH and 295 K. The dots represent experimental data for $A_{oligomer}/A_{H_2O}$, and the lines show model fits of $m_{oligomer}$ under different choices of reaction rate constants. (a) Reference case using the same rate constants as in the main manuscript: $k_{f,s} = 6 \times 10^{-3}\, kg\, mol^{-1} s^{-1}$, $k_{b,s} = 3 \times 10^{-4}\, s^{-1}$, $k_{f,b} = 1.8 \times 10^{-9} kg\, mol^{-1} s^{-1}$, $k_{b,b} = 9 \times 10^{-11} s^{-1}$, (b) $k'_{f/b,s} = 0.1 k_{f/b,s}$ and $k'_{f/b,b} = k_{f/b,b}$. (c) $k''_{f/b,s} = 10 k_{f/b,s}$ and $k''_{f/b,b} = k_{f/b,b}$. (d) $k'''_{f/b,s} = k_{f/b,s}$ and $k'''_{f/b,b} = 10 k_{f/b,b}$.

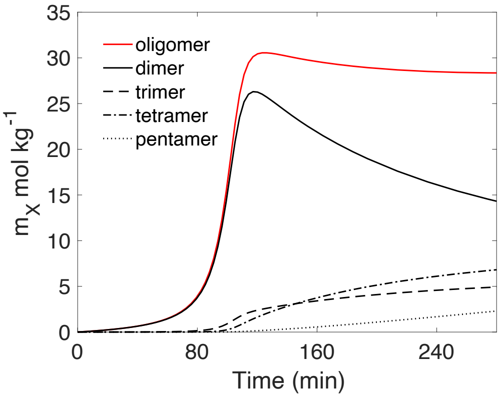

Figure S6: Time evolution of the concentrations of dimers (M_2), trimers (M_3), tetramers (M_4), pentamers (M_5), and their total (oligomers) in a droplet with an initial radius of 36 μm at RH=80%. The reaction rate constants used are the same as in Figure 3.

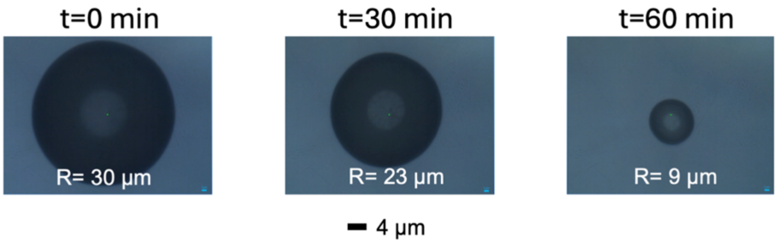

Figure S7: Bright-field images captured during micro-Raman spectroscopy during LA oligomerization.

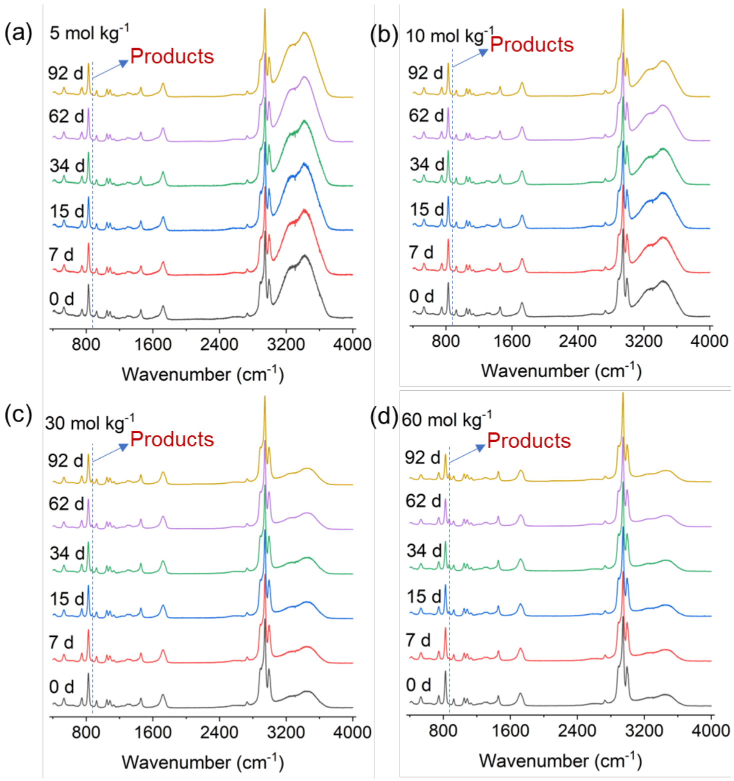

Figure S8. Raman spectra of LA bulk solutions with concentrations of (a) 5, (b) 10, (c) 30 and (d) 60 mol kg^{-1} in the dark from 0 to 92 days.

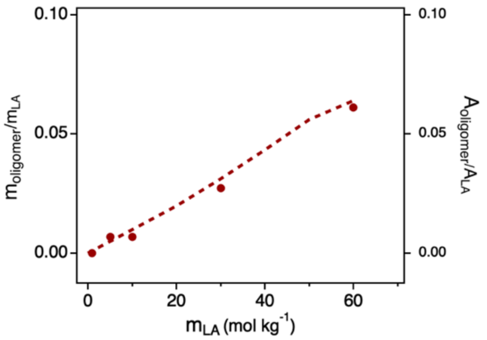

Figure S9: Changes in equilibrium oligomer-to-LA ratio as a function of m_{LA} in bulk solutions. Dashed lines represent model fits to the experimental data.

REFERENCES

(1) Szyszkowski, B. Experimentelle Studien über kapillare Eigenschaften der wässrigen Lösungen von Fettsäuren. Z. Phys. Chem. **1908**, 64, 385–414.

(2) Berg, J. C. An Introduction to Interfaces and Colloids: The Bridge to Nanoscience, 2nd ed.; World Scientific: Singapore, **2010**.

(3) Yang, S.; Li, M.; Wang, J.; Grassian, V. H.; Kumar, S.; Dutcher, C. S. Role of interfacial processes in accelerated reactions in nano- and microdroplets. J. Phys. Chem. A **2025**, *129*, 6424-6436.

(4) Bleys, G.; Joos, P. Adsorption kinetics of bolaform surfactants at the air/water interface. J. Phys. Chem. **1985**, *89*, 1027–1032.

(5) Fuller, E. N.; Schettler, P. D.; Giddings, J. C. New method for prediction of binary gas-phase diffusion coefficients. Ind. Eng. Chem. **1966**, *58*, 18–27.

(6) Pyda, M.; Czerniecka-Kubicka, A. Thermal properties and thermodynamics of poly(L-lactic acid). In Synthesis, Structure and Properties of Poly(L-lactic acid); Springer: Cham, **2017**; pp 153–193.
